# A novel asexual blood-stage malaria vaccine candidate: PfRipr5 formulated with human-use adjuvants induces potent growth inhibitory antibodies

**DOI:** 10.3389/fimmu.2022.1002430

**Published:** 2022-10-27

**Authors:** Eizo Takashima, Hikaru Nagaoka, Ricardo Correia, Paula M. Alves, António Roldão, Dennis Christensen, Jeffrey A. Guderian, Akihisa Fukushima, Nicola K. Viebig, Hilde Depraetere, Takafumi Tsuboi

**Affiliations:** ^1^ Division of Malaria Research, Proteo-Science Center, Ehime University, Matsuyama, Japan; ^2^ iBET, Instituto de Biologia Experimental e Tecnológica, Oeiras, Portugal; ^3^ Instituto de Tecnologia Química e Biológica António Xavier, Universidade Nova de Lisboa, Oeiras, Portugal; ^4^ Center for Vaccine Research, Statens Serum Institut (SSI), Copenhagen, Denmark; ^5^ Access to Advanced Health Institute, Seattle, WA, United States; ^6^ Vaccines, Sumitomo Pharma Co., Ltd., Osaka, Japan; ^7^ European Vaccine Initiative, UniversitätsKlinikum Heidelberg, Heidelberg, Germany; ^8^ Division of Cell-Free Sciences, Proteo-Science Center, Ehime University, Matsuyama, Japan

**Keywords:** asexual blood-stage malaria vaccine, PfRipr5, *Plasmodium falciparum*, adjuvant, Alhydrogel, GLA-SE, CAF01

## Abstract

PfRipr is a highly conserved asexual-blood stage malaria vaccine candidate against *Plasmodium falciparum*. PfRipr5, a protein fragment of PfRipr inducing the most potent inhibitory antibodies, is a promising candidate for the development of next-generation malaria vaccines, requiring validation of its potential when formulated with adjuvants already approved for human use. In this study, PfRipr5 antigen was efficiently produced in a tank bioreactor using insect High Five cells and the baculovirus expression vector system; purified PfRipr5 was thermally stable in its monomeric form, had high purity and binding capacity to functional monoclonal anti-PfRipr antibody. The formulation of purified PfRipr5 with Alhydrogel^®^, GLA-SE or CAF^®^01 adjuvants accepted for human use showed acceptable compatibility. Rabbits immunized with these formulations induced comparable levels of anti-PfRipr5 antibodies, and significantly higher than the control group immunized with PfRipr5 alone. To investigate the efficacy of the antibodies, we used an *in vitro* parasite growth inhibition assay (GIA). The highest average GIA activity amongst all groups was attained with antibodies induced by immunization with PfRipr5 formulated with CAF^®^01. Overall, this study validates the potential of adjuvanted PfRipr5 as an asexual blood-stage malaria vaccine candidate, with PfRipr5/CAF^®^01 being a promising formulation for subsequent pre-clinical and clinical development.

## Introduction

Three main malaria vaccine types have been considered to target the different life cycle stages of *Plasmodium falciparum*, namely, pre-erythrocytic vaccines, asexual blood-stage vaccines, and transmission-blocking vaccines. Last year, the World Health Organization (WHO) for the first time recommended the use of a pre-erythrocytic vaccine based on circumsporozoite protein (CSP), RTS,S/AS01, for the prevention of *P. falciparum* malaria in children living in regions with moderate to high transmission (1). However, in the RTS,S/AS01 phase 3 trial (2) the vaccine efficacy is modest, and efficacy against clinical malaria wanes more rapidly than efficacy against infection (especially in high transmission settings) due to higher levels of naturally acquired immunity by the blood-stage infection in the control cohort than the vaccine cohort (3). Therefore, to maintain blood-stage immunity an asexual blood-stage vaccine is considered an important addition to a pre-erythrocytic vaccine (4).

High polymorphism levels in *P. falciparum* asexual blood-stage malaria vaccine antigens often result in strain-specific immunity that hampers vaccine efficacy in the clinical trials (5). Thus, developing vaccines based on conserved antigens across multiple strains could be a more straightforward approach to attain high protective efficacy in the field (6). The PfRipr/PfCyRPA/Rh5 protein complex is considered to play one of the central roles in the sequential molecular events leading to *P. falciparum* merozoite invasion (7). Since all three subunit proteins are highly conserved and naturally acquired antibody responses in humans against each of them are associated with clinical protection against malaria (8–10), the PfRipr/PfCyRPA/Rh5 protein complex components are considered as promising asexual blood-stage vaccine candidates. A phase I/IIa trial of RH5.1, a recombinant protein-based antigen derived from Rh5, formulated with AS01B adjuvant, showed induction of antibodies in humans that can significantly reduce the growth of asexual blood-stage parasites *in vivo* following controlled human malaria infection (CHMI) using blood-stage parasites (11, 12), thus making Rh5 the leading asexual blood-stage vaccine candidate from the PfRipr/PfCyRPA/Rh5 complex. However, the RH5.1 vaccine-induced protection was modest and only led to a 1- to 2-day delay in time to diagnosis without sterile protection (11).

To develop an improved next-generation asexual blood-stage malaria vaccine targeting the PfRipr/PfCyRPA/Rh5 complex, PfRipr is one of the promising antigen targets because of the following accumulated evidence. Specific antibodies raised against recombinant PfRipr protein exhibited strain-transcending inhibition of *P. falciparum in vitro* growth (7). We have successfully expressed the cysteine-rich region of PfRipr (K279-D995 amino acids (aa)) using the wheat germ cell-free system (WGCFS) (13). Next to investigating the protective efficacy of the antibodies, we used an *in vitro* parasite growth inhibition assay (GIA), and demonstrated the significant GIA activity of anti-PfRipr antibodies using both homologous *P. falciparum* 3D7 and heterologous FVO strains *in vitro* (14). However, using PfRipr as vaccine target is challenging due to its large size (126-kDa in full length) and highly cysteine-rich nature (87 cysteine residues). To overcome this barrier, we expressed 11 truncated protein fragments derived from PfRipr spanning 152-aa to 215-aa each. We then immunized animals using each PfRipr fragment to generate fragment specific polyclonal antibodies. By GIA measurement of the antibodies, we identified PfRipr5 (aa C720-D934) as a protein fragment inducing the most potent growth inhibitory antibodies as comparable level to the antibodies against full-length PfRipr (15). In agreement with this finding, an independent report showed that an anti-PfRipr monoclonal antibody (mAb) with GIA activity recognized aa N816-L860, a part of PfRipr5 (16). Although a direct comparison of GIA with polyclonal anti-RH5.1 and anti-PfRipr5 antibodies has not been conducted yet, this previous study suggested that rabbit polyclonal antibodies raised against recombinant PfRipr proteins with Freund adjuvant would have a comparable or greater GIA activity than anti-Rh5 antibodies in four laboratory strains of *P. falciparum* (16). In addition, only one non-synonymous single-nucleotide polymorphisms with minor allele frequency 9.13% is found in PfRipr5 (A755G), as opposed to those found in RH5.1 (H148D, Y147H, S197Y, C203Y, and I140M); thus PfRipr5 is more conserved than RH5.1 (7). Therefore, PfRipr5 is regarded as a promising asexual blood-stage vaccine candidate antigen for next-generation asexual blood-stage and combination vaccines against *P. falciparum*.

Adjuvants play a key role to enhance the efficacy of weakly-immunogenic antigens and/or to induce appropriate immune responses (17). Since most of the subunit malaria vaccine antigens considered to date are weak immunogens, the choice of adjuvant is a critical component for malaria vaccine development (18). Aluminum-based adjuvants are considered the gold standard among the human applicable adjuvants thanks to their safety and track-record (19), but novel adjuvants might be a better choice for a malaria vaccine. Formulation of PfAMA1-DiCo [an asexual blood-stage vaccine candidate based on the three recombinant variants of *P. falciparum* apical membrane antigen 1 (AMA1)] and PRIMVAC and PAMVAC (two placental malaria vaccines based on the VAR2CSA protein) with a non-aluminum-based adjuvant, glucopyranosyl lipid adjuvant–stable emulsion (GLA-SE) were shown to be safe and well-tolerated, and induced higher levels of functional antibodies compared to aluminum-based adjuvant, Alhydrogel^®^ (20–22). GLA-SE is a TLR4 agonist with potential to enhance the Th1 cell-mediated cytotoxic T lymphocyte (CTL) response and shown to be safe and well tolerated in human subjects in multiple phase I clinical trials (18, 23). In addition to GLA-SE, CAF^®^01 is a novel two-component liposomal adjuvant system composed of a cationic liposome vehicle [dimethyldioctadecylammonium (DDA)] stabilized with trehalose 6,6-dibehenate (TDB), a synthetic variant of mycobacterial glycolipid cord factor which is recognized by the C-type lectin receptor MINCLE and has been shown to be safe in human trials (24, 25). The CAF^®^01 adjuvant has recently been tested in a phase 1/2a GMZ2 asexual blood-stage malaria vaccine clinical trial for the first time and the GMZ2/CAF^®^01 vaccine was well tolerated and immunogenic in humans (26). This CAF^®^01’s unique mode of action makes it an attractive candidate adjuvant for a future malaria vaccine.

In this study, we produced PfRipr5 antigen using insect cells and a baculovirus expression vector system, and performed a head-to-head comparison of its antigenicity when formulated with adjuvants for human use, specifically Alhydrogel^®^, GLA-SE, and CAF^®^01, as well as functional activity of the rabbit antibodies, to further advance the development of a PfRipr5-based malaria vaccine candidate.

## Materials and methods

### Production of PfRipr5

PfRipr5 recombinant protein was produced in a 50 L stirred-tank bioreactor (Sartorius, Göttingen, Germany) by infecting insect High Five cells (Invitrogen, Carlsbad, CA) at 2 ×10^6^ cell/mL with a recombinant baculovirus encoding *pfripr5* nucleotide sequence and His_6_-tag for purification, using a multiplicity of infection of 0.1 virus per cell, as described elsewhere (27). Cells were expanded by sub-culturing at 0.3-0.5 ×10^6^ cell/mL every 2-3 days when cell density reached 2-3 ×10^6^ cell/mL in Insect-XPRESS™ (Sartorius) and at 27°C, using shake-flasks (Corning, Corning, NY) of 500 mL (N-4 stage) and 2000 mL (N-3 stage), and stirred tank-bioreactors (Sartorius) of 2 L (N-2 stage), 10 L (N-1 stage) and 50 L (production stage, N). For shake-flask cultures, cells were maintained in a shaking incubator (Inova 44R – Eppendorf, Hamburg, Germany) set to 100 rotations per minute (rpm) and with 2.54 cm shaking diameter. For bioreactor cultures, pO_2_ was set to 30% of air saturation and was maintained by varying the agitation rate from 60 to 270 rpm and the percentage of O_2_ in the gas mixture from 0 to 100%, the gas flow rate was set to 0.01 volume per volume per minute (vvm).

### Purification of PfRipr5

Purification of secreted PfRipr5 was carried out on ÄKTA Explorer 100 systems (Cytiva, Tokyo, Japan) as described elsewhere (27). In brief, cell culture bulk was clarified using a Sartopore 2 30’’ 0.45 µm + 0.2 µm filter (Sartorius), loaded on a Histrap HP column (Cytiva), and protein was eluted with a linear Imidazole gradient. The eluate was concentrated using a Vivaflow 200 Hydrosart 10 kDa (Sartorius) and loaded into a Superdex 75 prep grade XK50/100 gel size-exclusion chromatography column (SEC) (Cytiva), from which fractions corresponding to monomeric PfRipr5 were collected. The collected fractions were loaded in a HiPrep desalting 26/10 column (Cytiva), the eluate was concentrated as mentioned above, and then sterile-filtered (0.2 μm). The final sample was formulated in 16 mM sodium phosphate buffer, 250 mM NaCl, at pH 8.0, aliquoted and stored at -80°C.

### Cell concentration and viability

Cell concentration and viability were assessed using a Cedex HiRes Analyzer (Roche, Basel, Switzerland).

### SDS-PAGE and western blot

SDS-PAGE and Western blot analyses were performed as described elsewhere (28). Reduced (R) samples were treated with NuPAGE Sample Reducing Agent 1× for 10 minutes at 70°C, whereas for non-reduced samples (NR) water was mixed instead. Then, both samples were run in the same gel (4-12% Bis_Tris, NuPAGE). For PfRipr5 identification by Western blot, anti-PfRipr5 antiserum previously generated in rabbits (15) was used (dilution 1:1000), and an anti-rabbit IgG antibody conjugated with alkaline phosphatase was used as secondary antibody (dilution 1:5000). Protein band detection was performed with NBT/BCIP 1-Step (Thermo Fisher Scientific, Waltham, MA). Densitometry analysis of SDS-PAGE gels was performed using Fiji software (29).

### Protein concentration

Protein concentration was determined by spectrophotometry at 280 nm using the mySPEC equipment (VWR, Radnor, PA).

### Dynamic light scattering

The size distribution of the purified PfRipr5 was analyzed by dynamic light scattering (DLS) on a Spectro Light 600 (Xtal Concepts, Hamburg, Germany).

### High performance liquid chromatography-Size-exclusion chromatography

Purified PfRipr5 protein was analyzed in a HPLC system equipped with Photodiode Array Detector (Waters, Milford, MA). Purified sample was loaded in a XBridge BEH 125 Å SEC 3.5 µm HPLC column (Waters), equilibrated in buffer containing 16 mM sodium phosphate, 250 mM NaCl, at pH 8.0, at a flow rate of 0.86 mL/min. Twenty micrograms of PfRipr5 was injected, and the eluted proteins were detected at 280 nm.

### Thermal shift assay

Purified PfRipr5 was mixed with a thermal shift dye (Thermo Fisher Scientific) in a MicroAmp™ EnduraPlate™ Optical 96-Well Fast Clear Reaction Plate with Barcode (Thermo Fisher Scientific) to a final volume of 20 µL (n = 2 measurements). Thermal shift assay was performed in a QuantStudio 7 Flex RealTime PCR System (Thermo Fisher Scientific), with excitation and emission wavelengths of 580 and 623 nm, respectively. Plates were heated from 25°C to 90°C (rate of 0.016°C per second) and fluorescence was measured. Results were analyzed using the Protein Thermal Shift™ Software V1.3.

### Surface plasmon resonance

Surface plasmon resonance (SPR) was carried out in Biacore X100 (Cytiva) as we previously described (27).

### Adjuvant and PfRipr5 formulation

The PfRipr5 antigen was formulated with three adjuvants compatible for human use. Alhydrogel^®^ was provided from Croda Denmark (Frederikssund, Denmark), CAF^®^01 was provided from Statens Serum Institut (SSI; Copenhagen, Denmark), and GLA-SE was provided from Access to Advanced Health Institute (AAHI; Seattle, WA). SSI and AAHI performed the compatibility studies using their routine assays to evaluate suitability of the adjuvants for formulation with PfRipr5. The PfRipr5 was diluted in 10 mM Tris buffer with 2% glycerol (pH=7.0) to the target concentration in each vaccine formulation. Five vaccine formulations (500 µL/dose) were devised, namely (i) Alhydrogel^®^ vaccine formulations containing Alhydrogel^®^ (5 mg/mL) and either 100 µg/mL (low dose) or 400 µg/mL (high dose) of PfRipr5, (ii) CAF^®^01 vaccine formulations containing CAF^®^01 (1250 µg/mL DDA and 250 µg/mL TDB), and either 100 µg/mL (low dose) or 400 µg/mL (high dose) of PfRipr5, and (iii) GLA-SE vaccine formulation containing GLA-SE (50 µg/mL) and 400 µg/mL (high dose) of PfRipr5. In human clinical trials, often 100 µg will be set as the highest dose. To evaluate a dose range, we used half (50 µg/dose) as low dose and twice (200 µg/dose) as high dose in this rabbit study. In the case of GLA-SE formulation study, if the higher antigen concentration formulation is compatible, we will be able to expect compatibility with the lower antigen concentration. Therefore, we only tested the high dose in the case of the GLA-SE formulation.

The antigen-adjuvant compatibility of all formulations was assessed *via* visual inspection and pH measurement at room temperature (RT) to mimic the on-site preparation for Alhydrogel^®^ or CAF^®^01 formulations one hour post formulation and for GLA-SE formulations at 0-, 4-, and 24-hour post formulation at RT and 5˚C. In addition, the interaction of the PfRipr5 antigen with Alhydrogel^®^ or CAF^®^01 was further evaluated by mixing the adjuvant with 100 µg/mL or 400 µg/mL of the PfRipr5 protein, centrifuging at 14,000 ×*g* for 15 minutes (Alhydrogel^®^ formulations) or 137,400 ×*g* for 30 minutes (CAF^®^01 formulations), and measuring non-adsorbed protein in the supernatant using bicinchoninic acid (BCA) protein assay kit (Thermo Fisher Scientific). In the case of GLA-SE formulation, antigen stability was assessed by sandwich-ELISA established in our recent study using functional mouse anti-PfRipr mAb (clone 29B11) as capture antibody (27). Because we have previously reported the binding of PfRipr5 with mouse anti-PfRipr5 mAb 29B11, shown to have a potent GIA activity (27), thereby being used as proxy for predicting its biological activity.

### Rabbit immunization

All rabbit immunizations were subcontracted to Kitayama Labes Co. Ltd (Ina, Japan), and the antisera were provided by the company. In brief, Japanese white rabbits (n=6 per group) were subcutaneously immunized with the PfRipr5 protein alone (50 µg/shot) or with PfRipr5 antigen (0, 50, and 200 µg/shot) formulated with the aforementioned adjuvants at the specific concentrations in 500 µL injection, twice at three-week intervals (Day 0 and Day 21). Antisera were collected two weeks after the last immunization (Day 35).

### Enzyme-linked immunosorbent assay

ELISA was conducted to measure anti-PfRipr5 rabbit antibody titer. The following buffers were used: (i) coating buffer, containing 50 mM sodium carbonate buffer pH 9.5, (ii) blocking buffer, containing 1% (w/v) bovine serum albumin (BSA) (nacalai tesque, Kyoto, Japan) in phosphate buffered saline (PBS), (iii) dilution buffer, containing 0.1% BSA in PBS, and (iv) stopping buffer, containing 1 M sulfuric acid (FUJIFILM Wako Pure Chemical, Osaka, Japan). Briefly, flat-bottom 96-well ELISA plates (Corning) were coated with 100 ng per well of PfRipr5 diluted with coating buffer. Plates were blocked with 300 μL/well of blocking buffer for 1 hour at 37˚ C. Five-times serial dilutions of each test rabbit serum starting from 1000-times dilution were prepared in dilution buffer. Diluted sera were added to antigen-coated wells in triplicate (50 μL/well) and incubated for 1 hour at 37°C. Plates were washed with washing buffer using a plate washer, and incubated with 100 μL/well of the goat anti-rabbit IgG conjugated with horseradish peroxidase (GE Healthcare, Chicago, IL) for 1 hour at 37°C . After washing, the substrate (0.1 mg/well of o-phenylenediamine, FUJIFILM Wako) diluted with 5 mM citric acid buffer pH 5.0 was added, and the plates were incubated at 37°C for 15 minutes. Reactions were stopped by adding 100 μL/well of stopping buffer. Absorbance was promptly measured at 492 nm using a Spectramax M3 microplate reader (Molecular Devices, Sunnyvale, CA). Reciprocal serum dilutions that gave a mean absorbance value of 0.5 at 492 nm were determined as the endpoint titers.

### Culturing *P. falciparum* and growth inhibition assay

Based on the highly conserved nature of PfRipr5 (16), we only used the *P. falciparum* 3D7 strain for the GIA to determine the functional activity of anti-PfRipr5 IgG. *P. falciparum* 3D7 strain was kindly provided by the National Institute of Allergy and Infectious Diseases (NIAID), and asexual stage of the parasite was cultured as described elsewhere (30). Total rabbit IgGs for GIA were purified from individual immune rabbit antisera with HiTrap protein G-Sepharose columns (GE Healthcare, Camarillo, CA) according to the manufacturer’s protocol. The GIA activity of the total IgGs from rabbit antisera against the PfRipr5 proteins was determined at 20 mg/mL final concentration over one cycle of *P. falciparum* 3D7 parasite replication. Parasitemia was determined by flow cytometry as described previously (15). Briefly, the parasite cultures were synchronized the day before the start of the GIA, so that the majority of parasites were at the late trophozoite-to-schizont stage at the start of the GIA. Twenty microliters of parasite-infected erythrocyte (pRBC) suspension (0.3% parasitemia and 2% hematocrit) and 20 µl of IgGs were added per well of half-area flat-bottom 96-well cell culture microplates (Corning) and gently mixed. For a control, 20 µl of culture medium was added to the pRBC suspension. Cultures were incubated at 37°C in humidified, gassed (90% N_2_, 5% O_2_, and 5% CO_2_), airtight boxes. After 25 hours of incubation, when most of the invading parasites had developed to the early trophozoite stage, the pRBC were pelleted by brief centrifugation (1,300 ×*g* for 5 min) and washed once in 100 µl PBS. The cells were then incubated with 50 µl of diluted (1:1,000 in PBS) SYBR green I (Invitrogen) for 10 min at RT and washed once in PBS. Parasitemia was measured by flow cytometry with a FACSCanto II (BD Biosciences, San Jose, CA) by the acquisition of 50,000 events per sample. Data were analyzed with FlowJo 9.1 software (Tree Star, Ashley, OR) by first gating for intact erythrocytes by side scatter and forward scatter parameters and subsequently determining the proportion of SYBR green I-positive cells. Rabbit IgGs obtained after immunization with Freund adjuvant formulated hexa-histidine-tagged glutathione S-transferase (His-GST) and region 3 to 5 of erythrocyte binding antigen 175 of *P. falciparum* (PfEBA175) (15) were used as a negative and positive controls, respectively. For each GIA, four independent experiments were carried out in triplicate to confirm the reproducibility and average GIA activities among four replicates obtained from each rabbit IgG were used for analyses.

### Statistical analysis

All statistical analyses were performed with GraphPad Prism (ver. 9.4.0) (GraphPad Software, San Diego, CA). Difference of the mean antibody titers and GIA activities among groups was tested by one-way ANOVA with Tukey’s multiple comparisons test. Pearson’s correlation coefficient between ELISA titers and GIA activities was calculated. P-values less than 0.05 were considered as statistically significant.

## Results

### PfRipr5 production

PfRipr5 was produced using insect High Five cells and the baculovirus expression vector system (IC-BEVS) at 50 L scale, and the quality of purified product was assessed by SDS-PAGE, western blot, Dynamic light scattering (DLS), High performance liquid chromatography-Size-exclusion chromatography (HPLC-SEC), Thermal shift assay (TSA) and Surface plasmon resonance (SPR).

**Figure 1:**
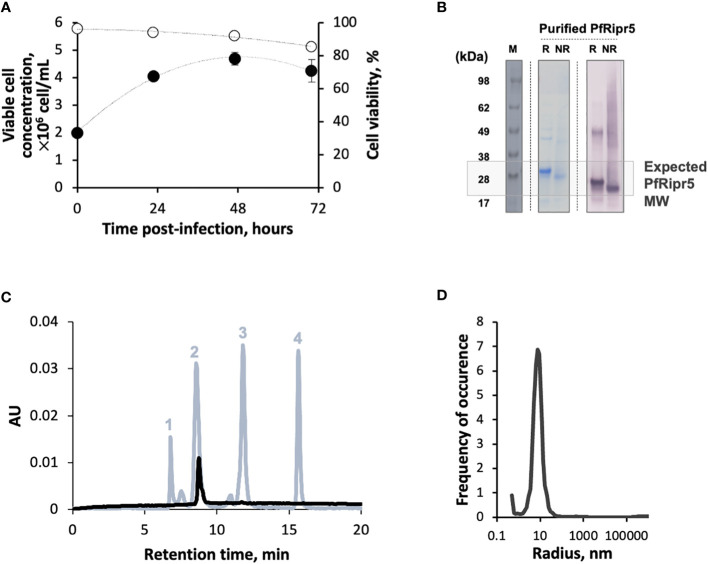
Production and characterization of PfRipr5. **(A)** Viable cell concentration (filled circles) and cell viability (open circles) throughout production process. **(B)** Identification of PfRipr5 by SDS-PAGE (middle panel) and Western blot (right panel) in purified sample. M denotes pre-stained protein standard SeeBlue^®^ Plus2, R denotes reduced sample, NR denotes non-reduced sample. **(C)** HPLC-SEC analysis of purified PfRipr5 protein (black line). The protein standard mix (grey line) used for estimation of PfRipr5 molecular weight was composed by (1) thyroglobulin (660 kDa), (2) ovalbumin (44.2 kDa), (3) ribonuclease A (13.7 kDa), and (4) uracil (112 Da). AU denotes absorbance unit (280 nm). **(D)** Size distribution profile of purified PfRipr5 assessed by dynamic light scattering (average of three measurements). A representative data of one biological replicate (n = 1) was shown.

Baculovirus infection kinetics followed a typical profile of a low MOI production process, i.e. High Five cell growth from 0-24 hours post-infection (hpi), onset of cell viability drop at 48 hpi, and culture harvest at 72 hpi (when cell viability reaches approx. 80%) (**Figure 1A**). Overall production yield was 0.8 mg/L, similar to previous reports (27). Bands corresponding to the expected molecular weight (Mw) size of monomeric PfRipr5 were identified by SDS-PADE and Western blot in the purified material (**Figure 1B**), with purity >85% (**Table 1**). HPLC and DLS data shows a single peak within the expected Mw (25-30 kDa) and radius (~10 nm), suggesting that purified PfRipr5 was mostly in monomeric form **(** **Figures 1C, D** **)**. In addition, the thermal stability of purified PfRipr5 (as assessed by thermal shift assay, melting temperature = 54 ± 2°C) as well as its ability to bind to the anti-PfRipr mAb 29B11 (as assessed by SPR, KD = 1.73 ± 1.6 × 10^-9^ M) were confirmed (**Table 1**).

**Table 1:** Characterization of purified PfRipr5.

Production yield, mg/L	Purity, %^*^	Melting temperature, °C^**^	KD, M^***^ (mAb 29B11)
0.8	> 85	54 ± 2	1.73 ± 1.6 × 10^-9^

^*^Purity assessed by densitometry analysis of SDS-PAGE.

^**^Melting temperature assessed by thermal shift assay.

^***^KD: equilibrium dissociation constant between PfRipr5 and mAb 29B11 assessed by surface plasmon resonance.

These data demonstrates that the purified PfRipr5 antigen herein produced in insect cells had high quality and thus was suitable for further formulation with adjuvants and animal immunizations.

### PfRipr5 formulation

The PfRipr5 antigen was formulated with three different adjuvants, Alhydrogel^®^, CAF^®^01, and GLA-SE, and the PfRipr5-adjuvant compatibility was confirmed following analyses optimized for each adjuvant.

Visual inspection of the PfRipr5 formulated with Alhydrogel^®^ or CAF^®^01 showed no changes as compared to Alhydrogel^®^ or CAF^®^01 alone, respectively. Likewise, the pH of the PfRipr5/Alhydrogel^®^ (7.2) and the PfRipr5/CAF^®^01 (7.0) was similar to that of each adjuvant alone, thus suggesting their chemical stability (**Table 2**). Quantification of PfRipr5 protein in the supernatant following centrifugation of these formulations was performed using BCA protein assay and showed that the concentration of non-adsorbed PfRipr5 in all the Alhydrogel^®^ and CAF^®^01 formulations was below the 25 µg/mL detection limit even for the high-dose PfRipr5 formulations, suggesting that all PfRipr5 antigen was adsorbed to both Alhydrogel^®^ and CAF^®^01 (**Table 2**). Visual inspection of PfRipr5 formulated with GLA-SE and GLA-SE alone was performed at 0, 4, and 24 hours post-formulation at 5°C and RT through assessment of color, opacity, and phase (**Table 3**); no visual variations of the 3 parameters were observed for any of the groups (PfRipr5/GLA-SE formulation and GLA-SE alone) or time points. Similarly, no major changes in pH were observed. Finally, sandwich-ELISA data suggests no apparent loss in binding affinity of the PfRipr5/GLA-SE formulation to the functional anti-PfRipr5 mAb 29B11 when compared to PfRipr5 antigen control, indicating that the desired conformation of a functional epitope in PfRipr5 recognized by the anti-PfRipr5 mAb 29B11 is maintained when mixed with GLA-SE throughout the assessed time (**Table 3**).

**Table 2:** Formulation of PfRipr5 with Alhydrogel^®^ and CAF^®^01.

	Alhydrogel^®^	100 µg/mL PfRipr5+ Alhydrogel^®^	400 µg/mL PfRipr5+ Alhydrogel^®^	CAF^®^01	100 µg/mL PfRipr5+CAF^®^01	400 µg/mL PfRipr5+CAF^®^01
Appearance*	Opaque suspension	Opaque suspension	Opaque suspension	Opaque suspension	Opaque suspension	Opaque suspension
pH**	7.2	6.5	7.3	7.0	6.5	7.2
Non-adsorbed PfRipr5***	–	Below detection level of 25 µg/ml	Below detection level of 25 µg/ml	–	Below detection level of 25 µg/ml	Below detection level of 25 µg/ml

*Appearance was determined by visual inspection.

**pH of the final vaccine formulation was measured, as this may be an indicator of chemical stability.

***Non-adsorbed PfRipr5 protein concentration in the supernatant after the centrifugation was determined by BCA.

**Table 3:** Formulation of PfRipr5 with GLA-SE.

	GLA-SE						GLA-SE + PfRipr5				
		5°C		RT				5°C		RT	
	T=0	T=4h	T=24h	T=4h	T=24h		T=0	T=4h	T=24h	T=4h	T=24h
Appearance*	White Opaque One Phase	No change	No change	No change	No change		White Opaque One Phase	No change	No change	No change	No change
pH**	6.36	6.36	6.34	6.35	6.30		6.91	6.87	6.79	6.84	6.84
Sandwich-ELISA***	No apparent loss of formulated PfRipr5 binding to mAb 29B11 in all the assay conditions in comparison with PfRipr5 antigen control (T=0)										

*Appearance was determined by visual inspection and was recorded at each time point according to three parameters: 1) color, 2) opacity, and 3) phase.

**pH of the final vaccine formulation was measured.

***Sandwich-ELISA was used to investigate the desired conformation of PfRipr5 is maintained when mixed with GLA-SE or not. Because we have previously reported the binding of PfRipr5 with mouse anti-PfRipr5 mAb 29B11, shown to have a potent GIA activity (27), thereby being used as proxy for predicting its biological activity.

RT, Room temperature.

These results indicate the acceptable compatibility of the PfRipr5 with all the tested adjuvants, and thus rabbit immunization using these vaccine formulations were performed.

### Immunogenicity of adjuvanted PfRipr5

**Figure 2:**
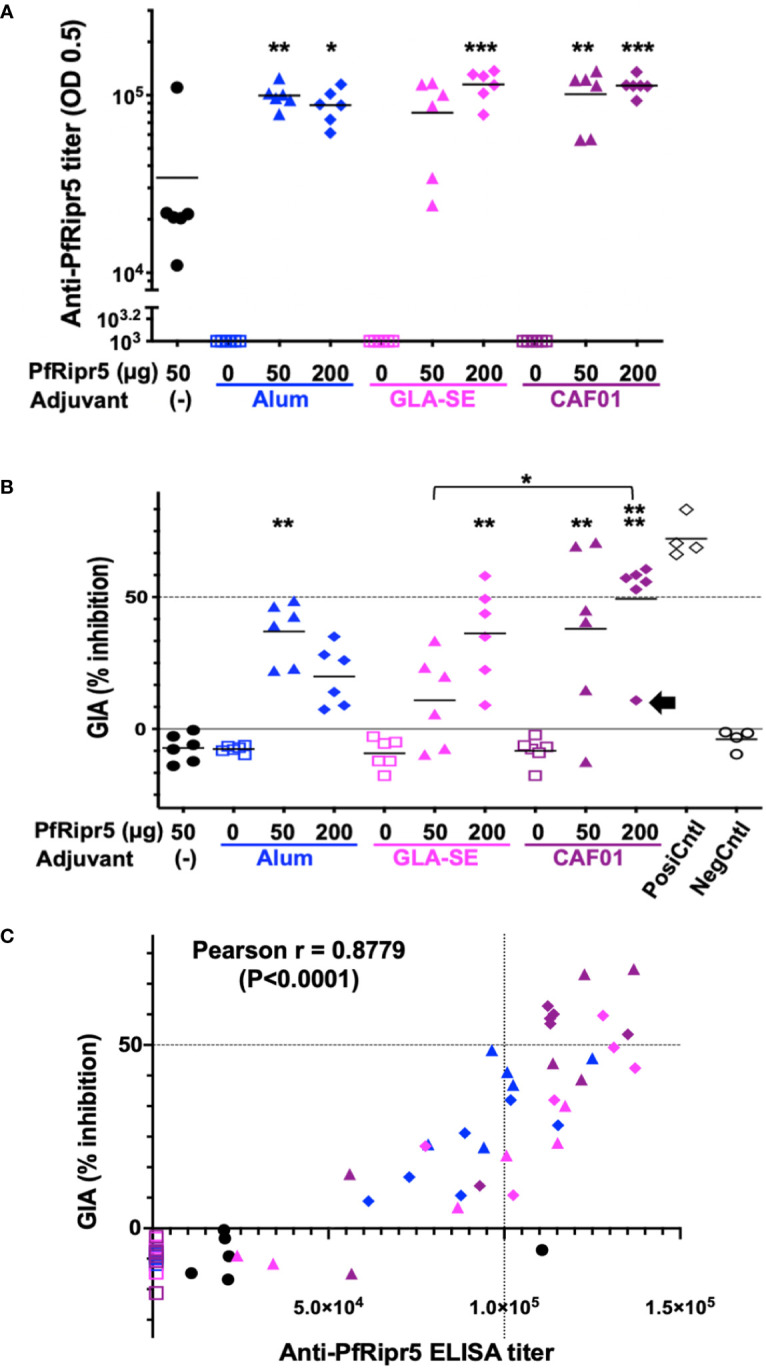
Immunogenicity of adjuvanted PfRipr5 and GIA activity of anti-PfRipr5 rabbit antibodies. PfRipr5 antigen only [(-); black filled circles], Adjuvant only (open squares), 50 µg dose of PfRipr5 (filled triangles), 200 µg dose of PfRipr5 (filled diamonds), formulation with Alhydrogel^®^ (Alum; blue), GLA-SE (GLA; magenta), and CAF^®^01 (CAF; purple). Difference of the mean antibody titers and GIA activities among groups was tested by one-way ANOVA with Tukey’s multiple comparisons test. P values less than 0.05 was considered as statistically significant difference and all the asterisks represent the significant difference against the PfRipr5 without adjuvant group unless otherwise indicated (*P<0.05; **P<0.01; ***P<0.001; ****: P<0.0001). **(A)** ELISA titer of anti-PfRipr5 antibodies. Reciprocal serum dilutions that gave a mean absorbance at 492 nm of 0.5 were determined as the endpoint titers. OD, optical density. **(B)** GIA activities of the total IgG purified from anti-PfRipr5 antibodies. For each GIA, four independent experiments were carried out in triplicate to confirm reproducibility and each data point represents average GIA of each rabbit IgG samples in four independent experiments. Rabbit IgGs immunized with Freund adjuvant formulated PfEBA175_region 3 to 5 (15) (PosiCntl; black open diamonds) and His-GST (NegCntl; black open circles) were used as a positive and negative controls, respectively. Black arrow indicates one outlier rabbit GIA data in the 200 µg PfRipr5/CAF^®^01 group. **(C)** Correlation between anti-PfRipr5 ELISA titers and GIA activities (n = 60). Pearson’s correlation coefficient was calculated as r = 0.8779 (P<0.0001).

Rabbits (n=6 per group) were subcutaneously immunized with the above-mentioned formulations. PfRipr5 alone (50 µg) induced significantly higher anti-PrRipr5 antibodies (Mean ELISA titer = 3.4 ×10^4^) than all the adjuvant alone groups (Alhydrogel^®^(Alum), GLA-SE (GLA), and CAF^®^01 (CAF) with 0 µg PfRipr5, P<0.05) suggesting that the PfRipr5 protein itself is immunogenic in rabbits (**Figure 2A**). Formulation of PfRipr5 with Alhydrogel^®^, GLA-SE, and CAF^®^01 induced statistically significant higher levels of antibodies in most low dose (50 µg) (Mean ELISA titers: Alum = 1.0 ×10^5^ (P<0.01); CAF = 1.0 ×10^5^ (P<0.01)) and in all high dose groups (200 µg)[Mean ELISA titers: Alum = 8.8 ×10^4^ (P<0.05); GLA = 1.2 ×10^5^ (P<0.001); CAF = 1.1 ×10^5^ (P<0.001)] than the PfRipr5 alone group, the exception being the low dose (50 µg) formulation with GLA-SE (Mean ELISA titer = 8.0 ×10^4^); no statistically significant difference was attained for the anti-PfRipr5 antibody titers between high and low dose within the adjuvant groups and across the adjuvant groups.

### GIA activity of the antibodies induced upon rabbit vaccination with adjuvanted PfRipr5

To address the functional activity of the rabbit antibodies induced upon rabbit immunization, *in vitro* GIA was conducted. The GIA activities of IgG induced by PfRipr5 GLA-SE and CAF^®^01 formulations were higher in the high dose (200 µg) (Mean %GIA: GLA = 36.2%; CAF = 49.4%) than in the low dose (50 µg) (Mean %GIA: GLA = 10.9%; CAF = 38%) groups, respectively (**Figure 2B**). In contrast, the GIA activity of IgG induced by PfRipr5 Alhydrogel^®^ formulation was higher in the low dose (50 µg) (Mean %GIA = 37%) than in the high dose (200 µg) (Mean %GIA = 19.9%) groups. In **Figure 2B**, the GIA activities of IgG induced by low dose (50 µg) PfRipr5/Alhydrogel^®^ formulation (P<0.01), high dose (200 µg) PfRipr5/GLA-SE (P<0.01), and low (50 µg)(P<0.01) and high dose (200 µg)(P<0.0001) PfRipr5/CAF^®^01 groups showed statistically significant levels of GIA activities compared to the PfRipr5 (50 µg)/no-adjuvant group. In addition, significantly higher GIA activity was also observed in the high dose (200 µg) PfRipr5/CAF^®^01 than low dose (50 µg) PfRipr5/GLA-SE groups. No statistically significant difference was attained for the other GIA activities between high and low dose within the adjuvant groups and across the adjuvant groups. However, importantly, eight rabbit IgG samples out of 60 samples demonstrated ≧50% GIA activities, belonging to the PfRipr5/CAF^®^01 groups (high dose: five rabbits; and low dose: two rabbits) and high dose PfRipr5/GLA-SE (one rabbit). Notably, if one outlier rabbit in the high dose (200 µg) PfRipr5/CAF^®^01 group was excluded from the analysis (**Figure 2B**, highlighted with black arrow), the mean GIA activity in this group (57.0%) would be comparable to that of the positive control group (Mean %GIA = 72.2%). Although PfRipr5 alone group induced anti-PfRipr5 antibody titers (**Figure 2A**), no GIA activity was observed (**Figure 2B**), thus demonstrating that the antigen-adjuvant formulations here devised are essential to obtain enough levels of anti-PfRipr5 antibodies for functional activity.

Overall, the CAF^®^01 adjuvanted PfRipr5 induced antibodies with the highest GIA activity thus being the most suitable formulation for subsequent pre-clinical studies.

### Correlation between ELISA titers and GIA activities

Anti-PfRipr5 antibody titers and GIA activities from 60 rabbits showed statistically significant positive correlation (Pearson’s correlation coefficient, r = 0.8779; P<0.0001) (**Figure 2C**). Especially, a group of IgG samples with higher GIA activities (≧50%) and higher antibody titers were mainly from rabbits immunized with CAF^®^01 formulations. In **Supplement Figure S1**, higher mean GIA activity was observed in the high dose (200 µg) PfRipr5/CAF^®^01 than high dose (200 µg) PfRipr5/GLA-SE group with similar levels of anti-PfRipr5 antibody titers, although statistical difference has not been reached significant. This observation indicates that higher IgG titer correlates with higher GIA activity and suggests that further efforts should focus on how to increase the anti-PfRipr5 specific antibody titers for the development of a potent PfRipr5-based vaccine formulation.

## Discussion

In this study, we tested head-to-head the adjuvanticity of PfRipr5 protein formulated with three adjuvants for human use (Alhydrogel^®^, GLA-SE, and CAF^®^01), to prioritize a PfRipr5-based malaria vaccine candidate for further advance its development. The PfRipr5 antigen herein produced was shown to be thermally stable in its monomeric form, having high purity and binding capacity to functional monoclonal anti-PfRipr antibody, thus assuring its quality for the subsequent use in animal experiments.

Polyclonal antibodies generated against PfRipr, PfCyRPA, or Rh5, were shown to inhibit merozoite invasion of *P. falciparum in vitro* (31–34), and thus the PfRipr/PfCyRPA/Rh5 complex components have been considered as promising asexual blood-stage vaccine candidates against *P. falciparum* malaria (35). Recently, Healer et al. (16) showed that rabbit antibodies raised against PfRipr resulted in significantly higher levels of GIA than those raised against either PfCyRPA or Rh5, whereas antibodies against the PfRipr/PfCyRPA/Rh5 complex showed the lowest level of inhibition, suggesting that the epitopes of some neutralizing antibodies were buried in the PfRipr/PfCyRPA/Rh5 ternary complex. These observations suggest that antibodies raised against PfRipr are more inhibitory than those raised against PfCyRPA, Rh5, or the PfRipr/PfCyRPA/Rh5 complex, and that vaccination with PfRipr alone might be sufficient to attain the desired parasite growth inhibition. In line with these findings, we have also previously shown strong growth inhibitory capacity of anti-PfRipr5 antibodies which also block PfRipr/Rh5 interaction, as well as that between PfRipr and its erythrocyte-surface receptor, SEMA7A (15). In the current study, we showed that all adjuvanted PfRipr5 formulations were stable during the period needed for vaccine administration. Furthermore, the observed immunogenicity, GIA activity of rabbit antibodies, and statistically significant positive correlation between antibody titers and GIA activities induced by PfRipr5 formulations suggest that PfRipr5 based vaccine development is feasible.

Recently, the first phase1/2a trial of Rh5 based blood-stage vaccine candidate, RH5.1, was conducted (11). In that study, the RH5.1/AS01B significantly reduced the *in vivo* parasite growth rate after blood-stage CHMI with *P. falciparum*. They also showed that *in vitro* GIA activity using purified human IgG significantly correlated with *in vivo* parasite growth rate. While the GIA measures a neutralization activity of purified IgG, the system lacks immune cells, complement, and other vaccine-induced antibody isotypes/subclasses. Therefore, in addition to the GIA activities induced by anti-PfRipr5 antibodies, it will still be worthwhile to investigate other immune pathways induced by PfRipr5 in first-in-human studies to evaluate the full potential of the PfRipr5 vaccine and its ability to induce *in vivo* efficacy and the capacity of natural infection to boost vaccine induced immune responses.

Some *P. falciparum* antigens are known to be highly immunogenic during natural infection (36, 37). In contrast, native PfRipr and Rh5 are weakly immunogenic antigens during natural infection (6, 10). Consistently, there was no evidence of natural boosting of anti-Rh5 antibodies in the primary CHMI using blood-stage *P. falciparum* challenge (11). Importantly, the current study shows that PfRipr5 antigen alone was immunogenic to rabbits without any adjuvant, although the generated antibodies could not induce significant GIA activities. Investigating whether anti-PfRipr5 antibody titers can be boosted when vaccinated humans receive multiple natural infections or CHMIs could be performed in future studies to understand the possibility of natural boosting of the vaccine-induced anti-PfRipr antibody titers.

Protein-based subunit malaria vaccine candidates that have been developed to date have poor immunogenicity. Therefore, targeted delivery of subunit vaccines *via* systems possessing adjuvant properties is of paramount importance (18) to ensure effective delivery and ability to increase protective immunity (38); the latter requires neutralizing antibodies (39, 40) and optimal Th1-mediated immunity (41). Several new-generation adjuvants in vaccine formulations have been approved for human use (42). A number of adjuvants, Alhydrogel^®^, CpG ODN, Montanide ISA, GLA-SE, GLA-LSQ, Adjuvant Systems, Matrix-M, and CAF^®^01 have been used for clinical trials assessing subunit malaria vaccines (18, 26, 43), and selection of the proper adjuvant needs to be tested antigen by antigen manner. In this study, we have explored adjuvanticity of the PfRipr5 antigen formulated with Alhydrogel^®^, GLA-SE, or CAF^®^01 because Alhydrogel^®^ is considered as the gold standard (19), GLA-SE showed better immunogenicity than Alhydrogel^®^ in some malaria vaccine clinical trials (20–22), and CAF^®^01 was previously used as novel adjuvant for a malaria vaccine candidate (26). In this study, the GIA activities of IgG induced by low dose Alhydrogel^®^ formulation, high dose GLA-SE, and low and high dose CAF^®^01 formulations showed statistically significant levels of GIA activities compared to the PfRipr5 no-adjuvant group. The high dose CAF^®^01 formulation showed the highest significance (P<0.0001) (**Figure 2B**). Furthermore, the number of rabbits with high GIA activities (≧50%) was highest in the PfRipr5/CAF^®^01 groups (high dose: 5/6 rabbits; and low dose: 2/6 rabbits). Finally, higher mean GIA activity was also observed in the high dose CAF^®^01 formulation than high dose GLA-SE group even with similar levels of anti-PfRipr5 antibody titers (**Supplementary Figure S1**). Although these results will be further strengthened in the future using larger number of animals to increase the statistical power, the PfRipr5/CAF^®^01 formulation was identified as the most promising vaccine candidate for further development because of its higher immunogenicity and induction of functional antibodies in rabbits. In fact, for the same antibody titers, the GIA activity of rabbit IgG induced by PfRipr5/CAF^®^01 formulation is higher than those induced by PfRipr5/GLA-SE formulations. These findings might be explained by the difference of antibody quality, such as epitope repertories and avidity. Additionally, the low dose of PfRipr5/Alhydrogel^®^ formulation showed higher antibody titers than high dose of Alhydrogel^®^ formulation. The immune mechanism underlining these results needs to be investigated further.

In general, it should be noted that it is difficult to predict adjuvanticity in humans from animal experiments. For instance, the use of a CAF^®^01-based formulation of GMZ2, one of the blood-stage malaria vaccine candidates, was superior to Alhydrogel^®^ in preclinical studies but not in human trials (26). In contrast, a chlamydia vaccine candidate CTH522 adjuvanted with CAF^®^01 had a better immunogenicity than Alhydrogel^®^ formulation in humans (25). Thus, adjuvanticity in humans is considered to be vaccine antigen dependent. Nonetheless, the fact that CAF^®^01 has the potential to induce potent inhibitory antibodies in rabbits supports further pre-clinical and clinical studies with this formulation.

In conclusion, we have identified that the GIA activity of rabbit IgG from PfRipr5/CAF^®^01 (200 µg) group was the highest among all the groups (approximately 50% inhibition), which is similar to the GIA activity of antibodies elicited against PfRipr5 with non-human applicable Freund’s adjuvant formulation (15). Based on the promising GIA results, the PfRipr5/CAF^®^01 formulation is suggested as the most suitable for subsequent pre-clinical and clinical development.

## Data availability statement

The original contributions presented in the study are included in the article/**Supplementary Material**. Further inquiries can be directed to the corresponding author.

## Ethics statement

The animal study was reviewed and approved by Kitayama Labes Co., Ltd. (Ina, Japan). All animal immunizations were commercially conducted at Kitayama Labes Co., Ltd. (Ina, Japan).

## Author contributions

ET, AR, AF, NV, HD and TT conceived and designed experiments. ET, HN, RC, DC, JG and AR conducted experiments. ET, AF, PA, AR and TT analyzed the data. ET, AF, NV, RC, HD and TT wrote the manuscript. All authors contributed to the article and approved the submitted version.

## Funding

This work was funded by the Japan-based Global Health Innovative Technology (GHIT) Fund (grant ID: T2018-151). This work was also supported in-part by JSPS KAKENHI (grant numbers: JP18H02651, JP20H03481, JP21H02724, JP21K06990, and JP21KK0138) and by a research fund from Sumitomo Pharma Co., Ltd. iNOVA4Health – UIDB/04462/2020 and UIDP/04462/2020, a program financially supported by Fundação para a Ciência e Tecnologia (FCT)/Ministério da Ciência, Tecnologia e Ensino Superior, through national funds is acknowledged. Funding from INTERFACE Programme, through the Innovation, Technology and Circular Economy Fund (FITEC), is gratefully acknowledged. This work was supported by FCT through the initiatives “Investigador FCT” Program (IF/01704/2014), Exploratory Research and Development Project (IF/01704/2014/CP1229/CT0001), and PhD fellowship (Ricardo Correia - SFRH/BD/134107/2017). The funders had no role in study design, data collection and analysis, decision to publish, or preparation of the manuscript.

## Acknowledgments

The Alhydrogel^®^ was a kind gift from Dr. Erik B. Lindblad, R&D Unit, Croda, Denmark. We thank the Japanese Red Cross Society for providing human erythrocytes and plasma for culturing *P. falciparum* parasites. We also thank Miwa Ochi, Yuiko Ogasawara, Ai Yanase, and Yusuke Hara for their technical assistance, and Flavia D’Alessio, Ezenwa James Onyemata, Robert Adamu Shey and Masauso Moses Phiri for project management support.

## Conflict of interest

AF is employed by Sumitomo Pharma Co., Ltd. and TT and ET are supported by a research fund from Sumitomo Pharma Co., Ltd. AF, TT, ET, and HN are inventors on patent PCT/JP 2017/040532 on a malaria vaccine antigen, PfRipr5. DC is co-inventor on patents on the cationic adjuvant formulations CAF. All rights have been turned over to SSI, which is a non-profit government research facility. JAG works for AAHI which has patent rights in the GLA-SE adjuvant formulation. These involvements did not influence the design of the study, the collection, analysis, access to, and interpretation of data, or the writing of the manuscript.

The remaining authors declare that the research was conducted in the absence of any commercial or financial relationships that could be constructed as a potential conflict of interest.

## Publisher’s note

All claims expressed in this article are solely those of the authors and do not necessarily represent those of their affiliated organizations, or those of the publisher, the editors and the reviewers. Any product that may be evaluated in this article, or claim that may be made by its manufacturer, is not guaranteed or endorsed by the publisher.

## Supplementary material

Supplementary Figure 1 Correlation between mean anti-PfRipr5 ELISA titers and mean GIA activities (n = 6 rabbits/group). PfRipr5 antigen only (black filled circle), Adjuvant only (open squares), 50 μg dose of PfRipr5 (filled triangles), 200 μg dose of PfRipr5 (filled diamonds), formulation with Alhydrogel^®^ (Alum; blue), GLA-SE (GLA; magenta), and CAF^®^01 (CAF; purple). Pearson’s correlation coefficient was calculated as r = 0.9438 (P<0.0001).
