# Supplementary figures and images for: A novel asexual blood-stage malaria vaccine candidate: PfRipr5 formulated with human-use adjuvants induces potent growth inhibitory antibodies
Source: Front Immunol. 2022 Oct 27;13:1002430. doi: 10.3389/fimmu.2022.1002430 (PMC9647036; doi:10.3389/fimmu.2022.1002430)

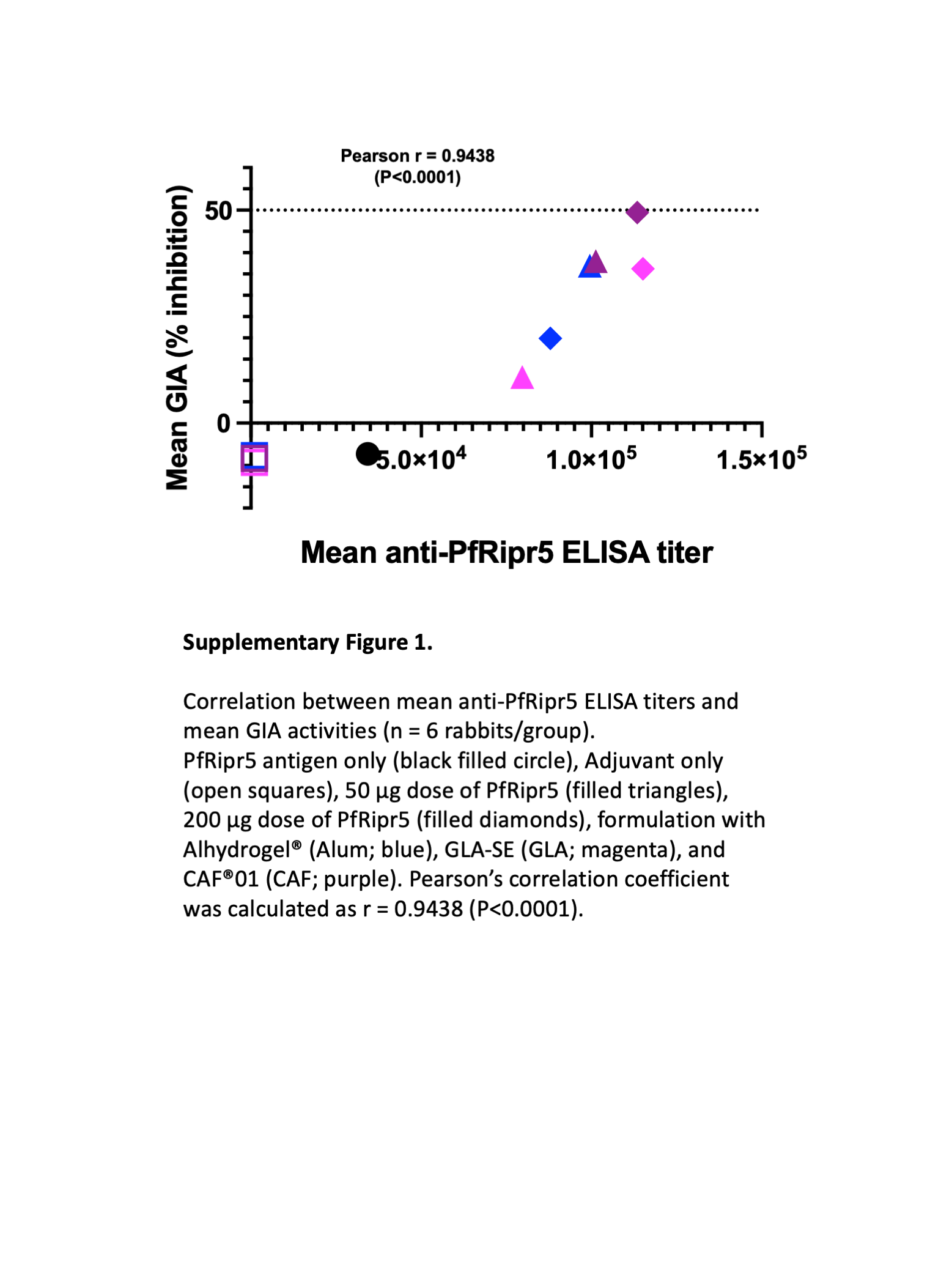

Supplement: Supplementary Figure 1 — Correlation between mean anti-PfRipr5 ELISA titers and mean GIA activities (n = 6 rabbits/group). PfRipr5 antigen only (black filled circle), Adjuvant only (open squares), 50 μg dose of PfRipr5 (filled triangles), 200 μg dose of PfRipr5 (filled diamonds), formulation with Alhydrogel® (Alum; blue), GLA-SE (GLA; magenta), and CAF®01 (CAF; purple). Pearson’s correlation coefficient was calculated as r = 0.9438 (P<0.0001). [file Image_1.tiff]
